# F-doped KMn_4_(PO_4_)_3_-based non-enzymatic electrochemical sensor for ascorbic acid and glucose: laboratory sensing and real-sample analysis

**DOI:** 10.1039/d6ra06582a

**Published:** 2026-09-30

**Authors:** Hekmat Alham, Sana Ahsen, Taqdees Tariq, Javeria Ahmed, Maria Khalid, Farhan Younas, Talha Nisar, Veit Wagner, Aansa Khaliq, Amin Badshah, Ali Haider

**Affiliations:** a Department of Chemistry, Quaid-i-Azam University Islamabad 45320 Pakistan aminbadshah@qau.edu.pk ahaider@qau.edu.pk; b H. E. J. Research Institute of Chemistry, International Center for Chemical and Biological Sciences, University of Karachi Karachi 75270 Pakistan; c Center for Interdisciplinary Research in Basic Sciences (CIRBS), International Islamic University Islamabad Islamabad Pakistan; d School of Science, Constructor University 28759 Bremen Germany; e Karlsruhe Institute of Technology (KIT), Institute for Applied Materials (IAM) Karlsruhe 76131 Germany; f Government Associate College for Women Mandi Shah Jewana Jhang Pakistan; g Pakistan Academy of Sciences 3-Constitution Avenue, Sector G-5/2 Islamabad 44000 Pakistan

## Abstract

The accurate detection of ascorbic acid and glucose demands electrode materials with high conductivity and fast electron transfer. Fluorine doping is an effective strategy to modulate the electronic structure and improve the conductivity of phosphate-based materials. Owing to these benefits, fluorine-doped potassium manganese phosphate (F-doped KMn_4_(PO_4_)_3_) was synthesized by the co-precipitation method and characterized using powder X-ray diffraction (PXRD) analysis, energy-dispersive X-ray spectroscopy (EDX), scanning electron microscopy (SEM), and X-ray photoelectron spectroscopy (XPS). The synthesized material was coated onto a nickel foam electrode, and its electrochemical sensing performance towards ascorbic acid and glucose was evaluated by electrochemical impedance spectroscopy (EIS), cyclic voltammetry (CV), and chronoamperometry. The fabricated electrode exhibited excellent sensing metrics: low detection limits (0.07 µM for ascorbic acid and 0.10 µM for glucose), fast response (2 s for ascorbic acid and 3 s for glucose), high sensitivity (800 µA mM^−1^ cm^−2^ for ascorbic acid and 1070 µA mM^−1^ cm^−2^ for glucose), and broad linear ranges (10 µM–14 mM for ascorbic acid and 50 µM–14 mM for glucose), with excellent reproducibility (RSD: 1.70% for ascorbic acid and 2.90% for glucose), repeatability, operational stability, and selectivity. In addition, the practical applicability of the sensor was confirmed through the quantification of ascorbic acid and glucose in real human blood serum samples, achieving maximum recoveries of up to 99% for both analytes.

## Abstract 2

Synthesized F-doped KMn_4_(PO_4_)_3_ showed high conductivity for electrochemical sensing. A modified Ni foam electrode detects ascorbic acid and glucose with LODs of 0.07 µM and 0.10 µM, respectively, 2–3 s response, and 99% recovery in serum.

## Graphical abstract

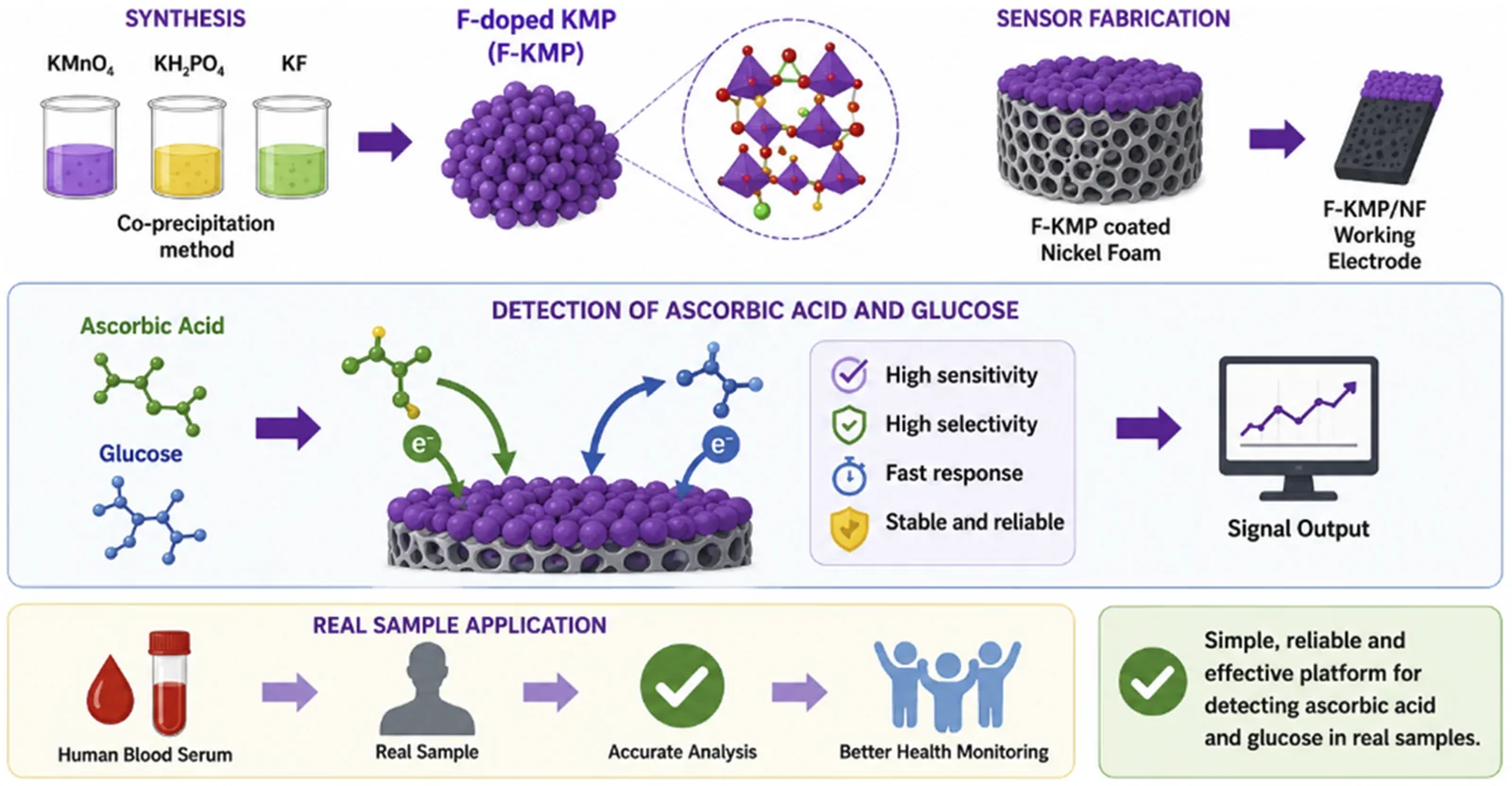

## 1. Introduction

Biomolecules are organic compounds essential for biological processes like growth, development and metabolism. They are mainly classified into macro- and micronutrients. Carbohydrates, proteins, lipids and nucleic acids are macronutrients, which are needed in large amounts for metabolic activities, while micronutrients, such as vitamins and minerals, are required in smaller amounts to maintain health.^1^ Research has further emphasized the importance of understanding the structural characteristics and biological functions of naturally derived biomolecules, including their potential health-related activities demonstrated through *in vitro* and *in vivo* studies.^2,3^ Besides their biological activities, they also serve as biomarkers for disease diagnosis.^4^ Two important biomolecules, namely, ascorbic acid and glucose, are considered to be the most important indicators for diagnosing and monitoring diseases like the common cold, ischemic injuries, Alzheimer's disease, and diabetes, making their sensitive detection essential for effective medical care.^5^ Diabetes is a chronic disease caused by the body not producing enough insulin or not using it effectively, leading to high blood glucose levels. This condition can cause serious health problems, including weight loss, kidney failure, heart disease, eye damage, and stroke, which may be life-threatening. The number of people with diabetes worldwide is rising, and it is expected to become the seventh leading cause of death by 2040.^6^ On the other hand, ascorbic acid is a water-soluble vitamin needed for various body functions. It helps the immune system, heals wounds, builds collagen, and protects cells from damage. Low ascorbic acid levels can cause diseases like scurvy, cancer, Alzheimer's, Parkinson's, and heart problems. However, excessive intake can cause stomach pain, kidney stones and diarrhea. The regular monitoring of blood glucose and ascorbic acid levels is crucial for early disease diagnosis and health assessment.^7^ Early disease diagnosis is fundamental for saving lives, improving treatment and increasing healthcare efficiency.^8^ Therefore, developing techniques for early biomolecule detection is crucial in the medical field.^9^

Analytical methods for the detection of ascorbic acid and glucose include liquid chromatography,^10,11^ spectrophotometry,^12,13^ fluorescence,^14,15^ colorimetry,^16^ and chemiluminescence.^17,18^ All these methods offer good accuracy and low detection limits but are often slow, costly and need skilled operators. Electrochemical techniques are other important methods that are widely used in food safety, environmental monitoring and healthcare.^19–21^ Approaches such as aptamer-based recognition,^22^ amplification-free CRISPR/Cas12a biosensing,^23^ and electrocatalysis-enhanced microfluidic arrays^24^ demonstrate the growing potential of advanced sensing platforms for rapid and sensitive analysis.

Electrochemical sensors are broadly classified into enzymatic and non-enzymatic forms. Enzyme-based sensors are highly selective due to enzyme specificity but suffer from low stability under certain conditions. In contrast, non-enzymatic electrochemical sensors are easy to prepare, highly stable against denaturation and storage, and provide efficient enzyme-like catalysis across a wide pH and temperature range.^25–27^ Furthermore, these electrochemical sensors are widely utilized due to their fast response time, low limits of detection, and high stability and sensitivity.^28,29^

Advancements in nanotechnology have introduced new opportunities to fabricate nanoengineered electrodes for sensing applications. For this purpose, different noble metals, such as Pt, Ag, and Au, are frequently used for the development of new non-enzymatic sensors.^29^ Noble metals are considered for this purpose due to their excellent corrosion resistance, high stability, and wide electrochemical windows. However, their high cost and low natural abundance limit their extensive applications.^30^ Extensive efforts have been directed towards developing highly active, affordable and durable electrocatalysts based on non-noble transition metals. For example, work on single-atom Fe sites supported by N-doped carbon has further highlighted the importance of synergistic active sites and electronic-structure engineering in improving catalytic performance.^31^ Various other electrode materials, such as transition metal borides, oxides, hydroxides, carbides, selenides, sulphides, nitrides, phosphides, and phosphates, have been identified as efficient and cost-effective alternatives for electrochemical applications.^32^ Among these, transition metal phosphates (TMPs) have been widely utilized for electrochemical applications due to their chemical stability, good electrochemical performance, electrical conductivity, non-toxic nature, abundant availability, and affordability.^33^ The most commonly employed metals among different TMPs are manganese (Mn), cobalt (Co), iron (Fe), nickel (Ni), zinc (Zn) and copper (Cu).^34^ TMPs often form layered structures with open frameworks that offer good conductivity. They contain metals with multiple oxidation states, ligands with flexible coordination modes and varied spatial arrangements, which enhance redox activity, rendering them highly suitable for electrode applications.^35^ Materials containing tetrahedral polyanion units (XO_4_), like those in phosphates, offer greater structural stability due to strong covalent P–O bonds.^36^

Currently, the Mn-based catalyst materials reported in the literature commonly show variations in factors such as valence state, structure, interlayer spacing, Mn vacancies, morphology, crystallinity, and surface state. These multiple interrelated parameters can collectively influence catalytic performance, making it challenging to directly pinpoint the exact cause of activity differences among various Mn-based systems. In KMnPO_4_, Mn and P are tetrahedrally coordinated, forming MnO_4_–PO_4_ six-membered rings that link into tunnel-like layers, with K^+^ ions inside. This layered arrangement enables faster transport and enhances activity.^37^ To further enhance ionic and electrical conductivity as well as stability, anion doping is considered an attractive strategy^38^ and is adopted by many researchers to redesign electrodes using anions, such as oxygen,^39^ halides and sulphides. Among these anions, fluorine doping has been found to improve the performance of the electrode materials by achieving good cycle stability and high-rate capability in layered-structure electrode materials.^40,41^ The fluoride ion (F^−^, size 133 pm) is almost the same size as the oxide ion (O^2−^, size 132 pm) and is thus capable of replacing oxygen atoms in crystals. F doping creates vacancies and electron defects that function as active sites for charge transfer, consequently boosting the material's electron flow and conductivity. In addition, fluorine is more electronegative than oxygen, forming stronger metal–F bonds than metal–O bonds, which makes the overall structure more stable.^42^ Fluorine doping can also reduce the band gap, thereby facilitating electron transition from the valence band to the conduction band and enhancing the material's electrical conductivity.^43^ Beyond conventional sensing approaches, materials engineering has demonstrated that tailoring electronic structure, active sites, and interfacial properties can provide new routes for improving the performance of catalytic and sensing platforms.^39,44,45^

In the present work, fluorine-doped potassium manganese phosphate (F-doped KMn_4_(PO_4_)_3_) was synthesized and coated onto a nickel foam substrate for the non-enzymatic electrochemical detection of ascorbic acid and glucose. Fluorine incorporation is hypothesized to modulate the electronic structure of KMn_4_(PO_4_)_3_, improve its electrical conductivity, and increase the availability of electrochemically active sites, thereby promoting faster charge transfer and enhanced electrocatalytic performance. Compared with the undoped material, the fluorine-doped electrode exhibited superior electrochemical activity, achieving high sensitivities and low detection limits towards both analytes. These findings demonstrate that fluorine doping is an effective strategy for optimizing phosphate-based electrocatalysts and highlight the potential of the F-doped KMn_4_(PO_4_)_3_ material as a sensitive and reliable platform for the determination of ascorbic acid and glucose in real samples.

## 2. Experimental

### 2.1. Chemicals and solvents

Manganese(ii) chloride tetrahydrate (MnCl_2_·4H_2_O) and sulphuric acid (H_2_SO_4_) were purchased from Merck (Germany). Dipotassium hydrogen phosphate (K_2_HPO_4_), potassium fluoride (KF), *N*-methyl-2-pyrrolidone (NMP), polyvinylidene difluoride (PVDF), ethanol (AnalaR) and acetone were obtained from Sigma-Aldrich (USA). All chemicals were used as obtained. Required standard solutions were prepared in distilled water. Nickel foam sheets were acquired commercially.

### 2.2. Synthesis

For the synthesis of the F-doped KMn_4_(PO_4_)_3_ material, 0.98 g of MnCl_2_·4H_2_O and 9.76 g of K_2_HPO_4_ were dissolved in a mixed solvent containing 50 mL of acetone and 150 mL of distilled water. The mixture was agitated for 20 minutes. Then, 0.21 g of KF was added as a source of the fluorine dopant and stirred for 24 hours at room temperature. The precipitate obtained was gathered by centrifugal washing with distilled water, followed by oven drying at 60 °C for 12 hours. The obtained white solid was ground into a fine powder using a mortar and pestle. Eventually, the powder was calcined at 300 °C for 1 hour in a muffle furnace to obtain the F-doped KMn_4_(PO_4_)_3_ material. A similar procedure was adopted for the synthesis of undoped KMn_4_(PO_4_)_3_ without the addition of KF for comparison purposes. Fig. 1 shows the stepwise experimental procedure for the synthesis of the materials.

**Fig. 1:**
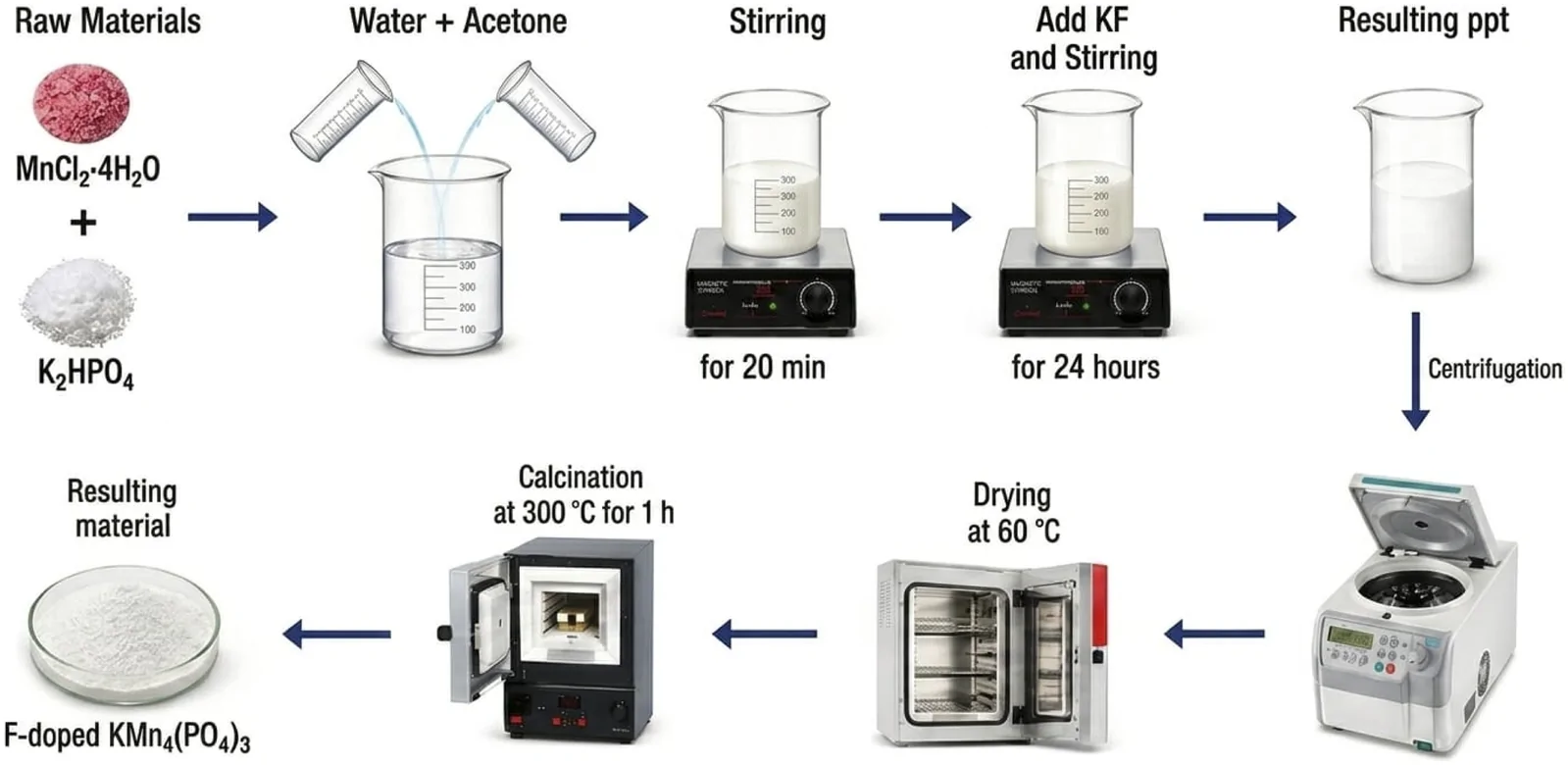
Schematic of the synthesis of the F-doped KMn_4_(PO_4_)_3_ material.

### 2.3. Pretreatment of nickel foam

Before fabrication, the commercially purchased nickel foam was cleaned to remove any organic impurities and inorganic oxide layers. First, the foam pieces (1 cm^2^ × 1 mm) were cleaned by sonication in acetone for 10 minutes to get rid of organic contents. Thereafter, they were treated with a 1 : 3 ratio of sulphuric acid and distilled water for 10 minutes to remove metal ions and oxide layers, followed by sonication in distilled water to remove acid impurities. After that, they were sonicated in ethanol for 5 to 10 minutes to get rid of any leftover impurities. Finally, the cleaned nickel foam was dried in an oven at 70 °C for 12 hours.

### 2.4. Electrode fabrication

To fabricate the working electrode, the synthesized material was mixed with a PVDF binder in a 9 : 1 ratio, followed by the addition of a few drops of NMP to form a thick liquid slurry (catalytic ink). This slurry was spread evenly onto activated nickel foam pieces and kept in an oven at 70 °C overnight before electrochemical tests.^43^

### 2.5. Characterization

Powder X-ray diffraction (PXRD) was performed using a PANalytical X'Pert system with a copper Kα source. The data were collected at a scan rate of 5° per minute, in the 2*θ* range of 10° to 80°. Elemental composition was determined by energy-dispersive X-ray (EDX) analysis, and the morphology of the material was examined using scanning electron microscopy (SEM), which was performed on an FEI NOVA SEN 230 microscope combined with a Bruker EDX system at 3 kilovolts.

To prevent surface-charge accumulation during X-ray photoelectron spectroscopy (XPS) analysis, the sample was affixed to the holder using conductive adhesive. Then, the material was placed within an ultra-high vacuum enclosure housing the photoelectron spectrometer that has a hemispherical detector and X-ray gun for magnesium and aluminum sources, positioned at a 45° angle. In this study, magnesium K-α X-rays with an energy of 1253.6 eV were used. The analyzer was set to pass 50 eV of energy with a chamber pressure of about 1 × 10^−7^ pascal, and the data were interpreted through the CASA XPS program. The background signals were removed using Shirley's method. The peaks were fitted using a simple shape called a Voigt function. For certain peaks (doublets), the width was kept the same for both, and the intensity ratio between the two components (2p_3/2_ and 2p_1/2_) was fixed at 0.5.

### 2.6. Electrochemical measurements and analyses

A Gamry Interface 1010E system was used for electrochemical measurements and analyses. A three-electrode setup was employed, where the working electrode was the fabricated nickel foam, the counter electrode was a Pt sheet (5 × 5 mm), and the reference electrode was Ag/AgCl; a 0.1 M KOH solution was used as the electrolyte. The electrocatalytic behavior of the fabricated electrodes for detecting analytes (ascorbic acid and glucose) was studied by CV. The voltage was scanned between −0.4 and 0.4 V for ascorbic acid and 0 and 0.8 V for glucose at a scan rate of 6 mV s^−1^. For amperometric measurements, the current responses to ascorbic acid and glucose were recorded by incremental addition of ascorbic acid and glucose while stirring continuously at specific potentials of 0.15 V and 0.60 V, respectively.

### 2.7. Ethical statement

All experiments were performed in compliance with the Declaration of Helsinki and were approved by the Ethics Committee at the International Islamic University, Islamabad, Pakistan, under reference no. IIU/ORIC/Bioethics/110. Informed consent was obtained from the human participants of this study.

## 3. Results and discussion

### 3.1. Structure and morphology of the F-doped KMn_4_(PO_4_)_3_ material

The structural behavior of the F-doped KMn_4_(PO_4_)_3_ material was examined using PXRD patterns. Based on the diffraction patterns, the crystallographic structure was determined to be orthorhombic. The PXRD patterns of the synthesized material display well-defined peaks at 2*θ* values of 11.19°, 14.80°, 27.42°, 29.16°, 32.77°, 34.74°, 40.41°, and 42.10°, as mentioned in Fig. 2(a). The diffraction peaks at the given 2*θ* values correspond to the (020), (110), (200), (220), (202), (061), (071), and (233) planes, respectively. All these planes are precisely matched with JCPDS card no. 00-039-0177, with a slight shift in the peak position, which can be attributed to fluorine doping into the crystal lattice.^46–48^ This agrees with previous reports, where substitution of O^2−^ by F^−^ leads to slight peak shifts due to their nearly identical ionic radii while preserving the crystal structure.^49,50^

**Fig. 2:**
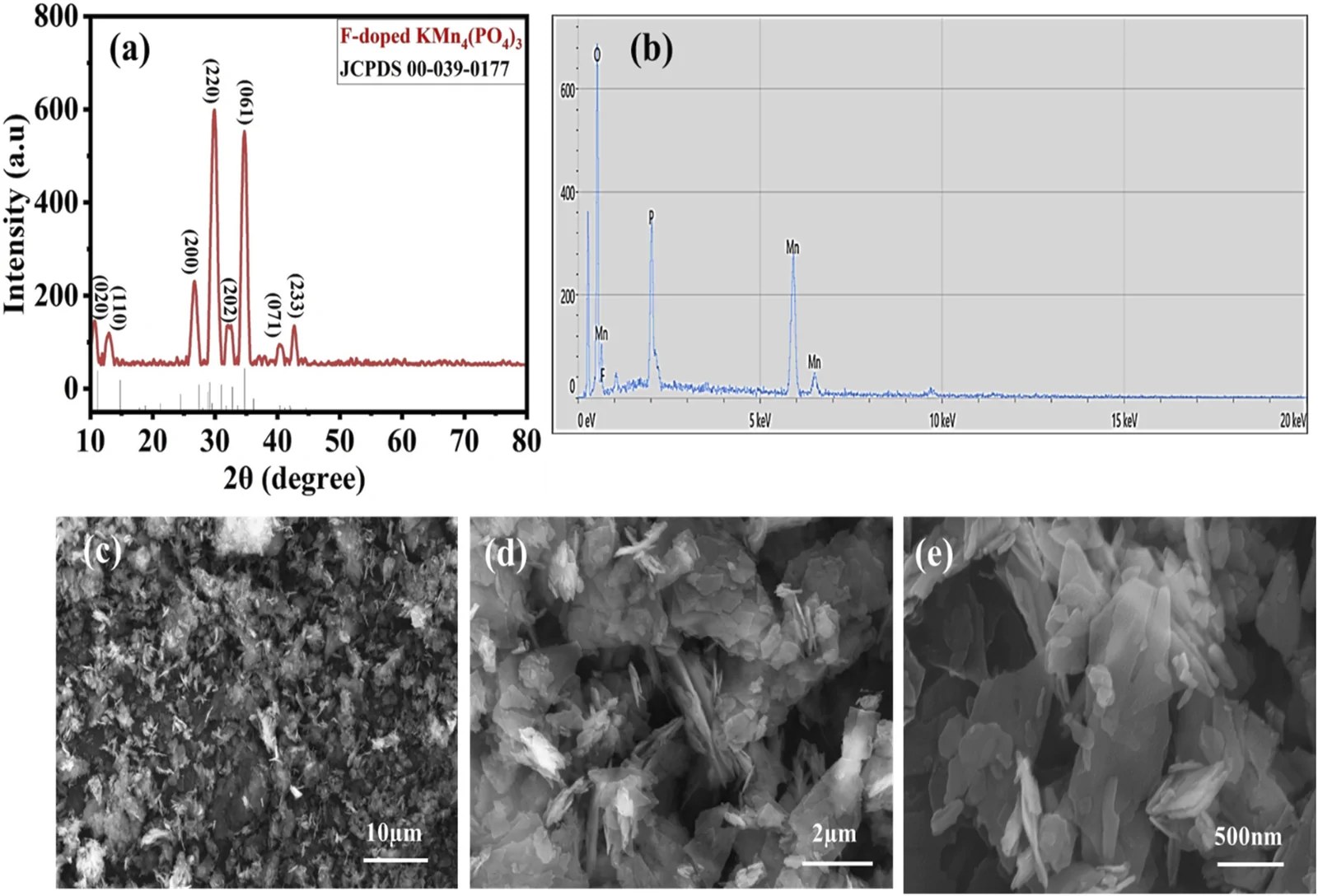
(a) PXRD pattern of the F-doped KMn_4_(PO_4_)_3_ material. (b) EDX spectrum of the F-doped KMn_4_(PO_4_)_3_ material. (c–e) SEM images of the F-doped KMn_4_(PO_4_)_3_ material under different magnifications.

The elemental composition of the F-doped KMn_4_(PO_4_)_3_ material was determined through EDX analysis. The EDX spectrum shows characteristic X-ray peaks of the constituent elements, which confirms the synthesis of the F-doped KMn_4_(PO_4_)_3_ material. Fig. 2(b) shows the EDX spectrum of the F-doped KMn_4_(PO_4_)_3_ material. The scanning electron micrograph of the F-doped KMn_4_(PO_4_)_3_ material displayed a flaky sheet morphology, showing thin, crumpled layers that are irregularly stacked and form a porous cluster network. At low magnification (10 µm scale bar), as shown in Fig. 2(c), the surface appears highly agglomerated with a rough texture. At higher magnification (2 µm scale bar), as shown in Fig. 2(d), the morphology becomes clearer, revealing flake-like and plate-shaped structures that are irregularly stacked. Further magnification (500 nm scale bar) in Fig. 2(e) confirms the presence of thin layered nanosheets with wrinkled surfaces and jagged edges, suggesting a two-dimensional growth behavior. This layered and porous architecture presented a high specific area and exposed active edge sites, which are favorable for enhanced electrochemical and catalytic performance.

The oxidation state of manganese ions in F-doped KMn_4_(PO_4_)_3_ was investigated by XPS analysis. The survey spectrum provides an overview of the synthesized material in Fig. 3(a), while the high-resolution spectrum confirms the specific oxidation state of the constituent elements. The high-resolution Mn 2p spectrum shows two major peaks at 641.8 and 653.8 eV, which can be attributed to Mn 2p_3/2_ and Mn 2p_1/2_, respectively, as shown in Fig. 3(b). The spin–orbit splitting of 12 eV for the Mn 2p doublet indicates an oxidation state of Mn(ii).^51,52^ The Mn 2p spectrum also reveals an additional minor Mn^3+^ contribution, where the higher-binding-energy components at approximately 644.3 and 656.1 eV are attributed to Mn^3+^, as shown in Fig. 3(b). Thus, the XPS results indicate that Mn^2+^ remains the dominant oxidation state, accompanied by a smaller fraction of Mn^3+^. The presence of Mn^3+^ can be rationalized by the charge compensation associated with F^−^ incorporation. The substitution of O^2−^ by F^−^ produces a local charge imbalance, which can be compensated in part by the oxidation of neighboring Mn^2+^ to Mn^3+^. The observed Mn^3+^ component is considered consistent with the electronic/charge-compensation effect induced by fluorine incorporation rather than a change of the fundamental KMn_4_(PO_4_)_3_ phase stoichiometry. Similar formation of higher-valent Mn species through fluorination and anion substitution has been reported for fluorinated Mn-containing materials.^53^ The high-resolution P 2p XPS spectrum mentioned in Fig. 3(c) was deconvoluted using a single component at 133.6 eV, corresponding to phosphorus in the synthesized material.^52^ Furthermore, the O 1s XPS spectrum appears in Fig. 3(d), revealing a metal–oxygen linkage through the deconvoluted component at 530.0 eV.^52^ A well-defined peak of fluorine (F 1s) indicates its successful doping into the KMn_4_(PO_4_)_3_ matrix, shown in both the survey and high-resolution spectra (Fig. 3(e)). Thus, the XPS analysis affirms successful fluorine doping into the potassium manganese phosphate matrix.

**Fig. 3:**
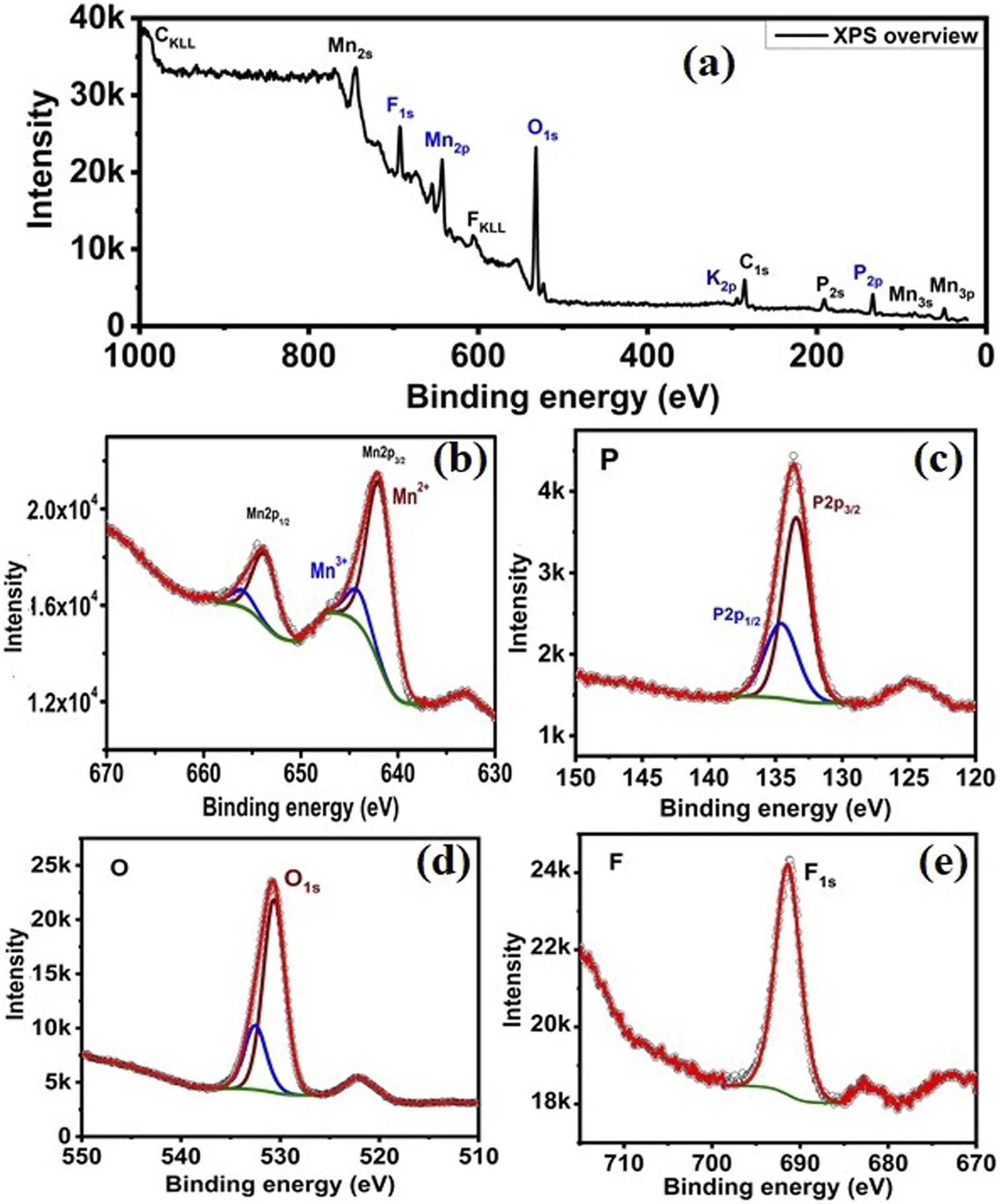
(a) XPS survey spectrum of the F-doped KMn_4_(PO_4_)_3_ material. High-resolution XPS spectra showing the oxidation states and chemical bonding environments of (b) Mn 2p, (c) P 2p, (d) O 1s and (e) F 1s.

### 3.2. Electrochemical studies

The electrochemical performance of the electrodes was evaluated using EIS, CV, and chronoamperometry techniques. EIS readings were taken in the frequency range of 0.1 Hz to 100 kHz using a 10 mV AC amplitude. For CV, the voltage range was −0.4 to 0.4 V for ascorbic acid and 0 to 0.8 V for glucose. In chronoamperometry, a fixed voltage of 0.15 V was used for ascorbic acid detection and 0.60 V for glucose detection.

EIS measurements were performed to estimate the charge-transfer efficiency of the fabricated electrodes. EIS provides valuable information about electrochemical system behavior and impedance responses.^54^ The obtained Nyquist plots compare sensor materials in 0.1 M KOH, measured from 0.1 Hz to 100 kHz with a small AC signal of 10 mV, as shown in Fig. 4. The diameter of the semicircle in the Nyquist plot corresponds to the charge-transfer resistance,^55^ which reflects the efficiency of the catalytic system in electrochemical sensing applications. The EIS results demonstrate that after F doping, the charge-transfer resistance of KMn_4_(PO_4_)_3_ decreases, which supports the superior catalytic activity of the F-doped KMn_4_(PO_4_)_3_ material. Fluorine possesses strong electronegativity, which allows it to pull electron density from surrounding atoms. This process creates electron-deficient regions or vacancies that act as active sites for charge transfer, thereby improving electronic conductivity. In addition, fluorine incorporation can modify the electronic band structure of the material, often leading to a reduction in the band gap. A smaller band gap makes it easier for electrons to move from the valence band to the conduction band, resulting in enhanced overall electrical conductivity.^43,56,57^

**Fig. 4:**
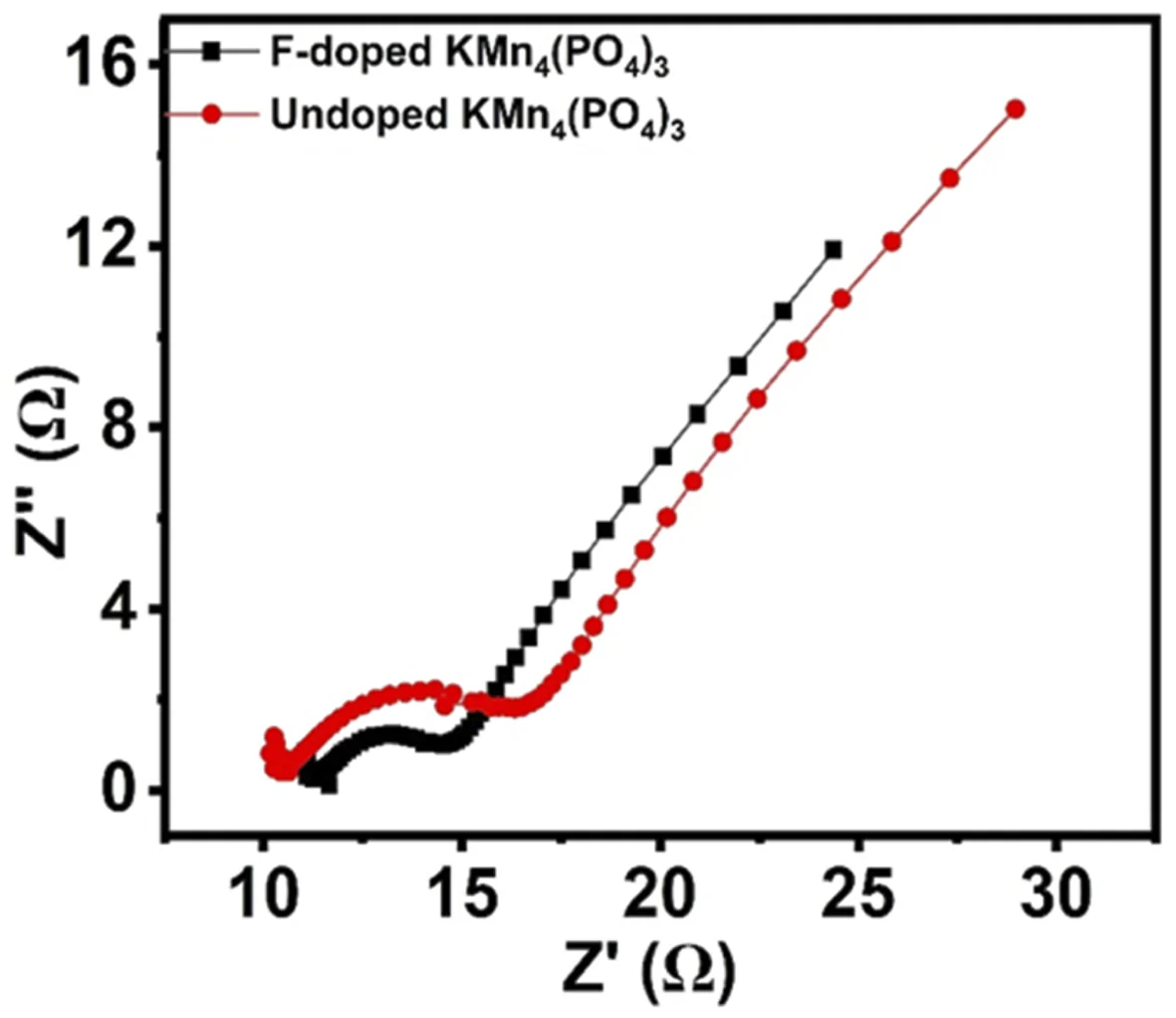
Overlaid Nyquist plots of undoped and F-doped KMn_4_(PO_4_)_3_.

Fig. 5(a and b) shows the electrochemical response of the F-doped KMn_4_(PO_4_)_3_ sensor for ascorbic acid and glucose (both 5 mM), respectively, in three different electrolytes: 0.1 M KOH, 0.1 M NaOH, and 0.1 M PBS, at a scan rate of 6 mV s^−1^. The sensor exhibited the highest current response in KOH for both analytes. This superior performance arises from the weaker hydration shell and higher ionic mobility of K^+^ compared to Na^+^, which enable faster charge transfer and more efficient movement of OH^−^ ions to the electrode surface. The rapid diffusion of OH^−^ in alkaline media further accelerates ascorbic acid oxidation.^43,58^ In contrast, PBS displays lower activity. This is due to the slow movement of bulky and highly hydrated phosphate ions, which limits their mobility and results in slower charge transfer compared to OH^−^ in alkaline solutions.^59^ Together, mobile K^+^ ions and an abundance of OH^−^ in KOH create ideal conditions for fast redox reactions. This boosts the electrocatalytic performance of the sensor.

**Fig. 5:**
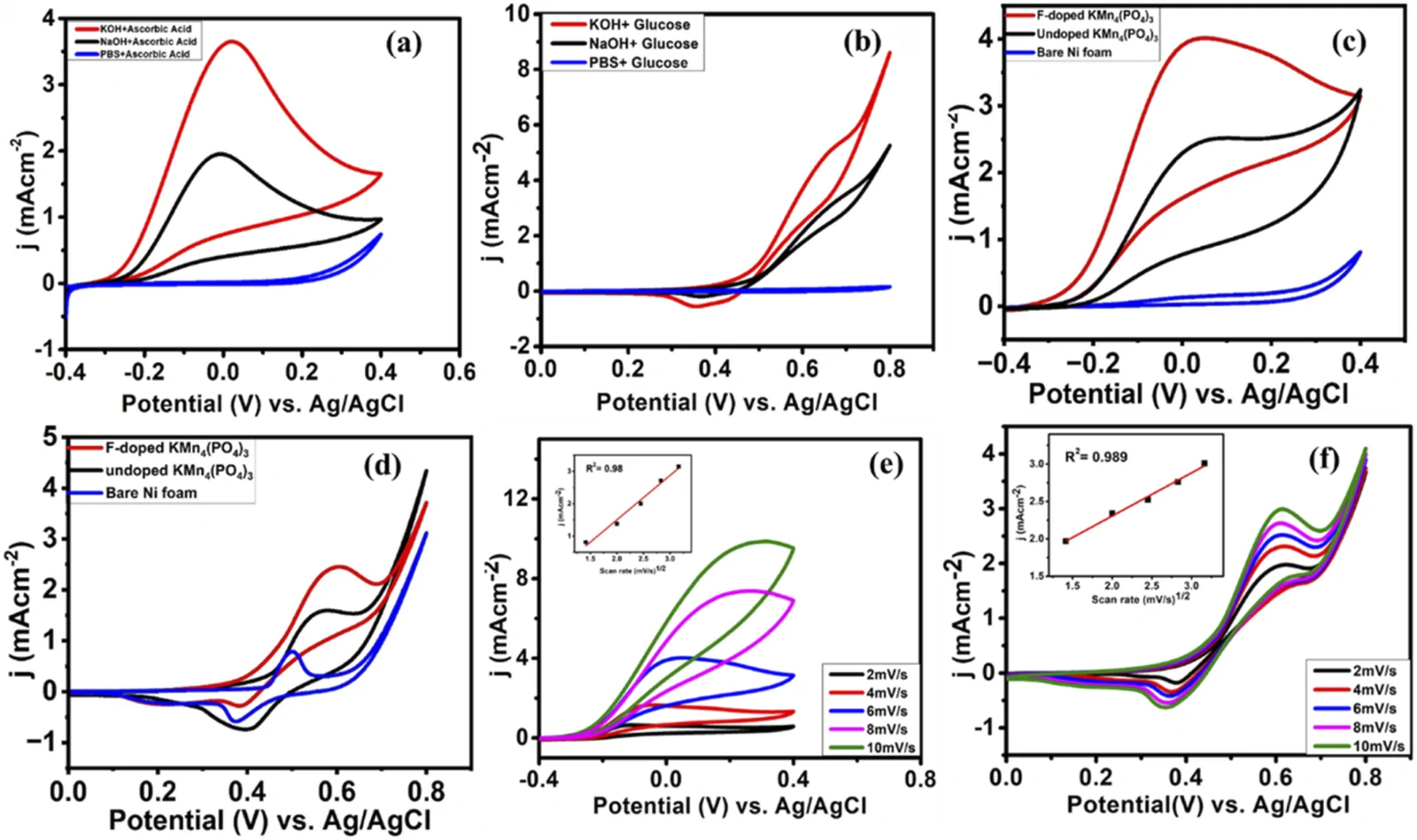
Electrochemical evaluation of F-doped KMn_4_(PO_4_)_3_. Comparison of the performance of F-doped KMn_4_(PO_4_)_3_ in different electrolytes with 5 mM (a) ascorbic acid and (b) glucose at 6 mV s^−1^. CV comparison of different sensors in 0.1 M KOH with 5 mM (c) ascorbic acid and (d) glucose at 6 mV s^−1^. CV curves in 0.1 M KOH at varying scan rates (2 to 10 mV s^−1^) with 5 mM (e) ascorbic acid and (f) glucose, with the insets showing the peak currents *vs.* square root plots of the scan rate, demonstrating a diffusion-controlled process.

The F-doping concentration was systematically optimized, and the electrochemical response increased with F content up to 10%, while further F doping resulted in a decrease in response. The electrochemical behavior of the optimized F-doped KMn_4_(PO_4_)_3_ for ascorbic acid as well as glucose was studied using cyclic voltammetry. Fig. 5(c and d) compares cyclic voltammograms of the bare nickel foam, undoped KMn_4_(PO_4_)_3_, and optimized F-doped KMn_4_(PO_4_)_3_ towards both ascorbic acid and glucose, respectively, at a concentration of 5 mM, in 0.1 M KOH at a scan rate of 6 mV s^−1^. F-doped KMn_4_(PO_4_)_3_ delivers the highest oxidation current density within specific potential windows for ascorbic acid and glucose detection, establishing it as an optimal non-enzymatic electrochemical sensor material. F doping provides oxygen vacancies, which help to provide more active sites. This accelerates the reaction kinetics, thus improving electrocatalytic activity.^57,60^ Furthermore, a comparison of the glucose detection performance using bare Ni foam, binder-coated Ni foam, and undoped and optimized F-doped electrodes in the presence of the same 5 mM glucose concentration is provided in Fig. S1. The enhanced current response is mainly attributed to glucose oxidation rather than the Ni(OH)_2_/NiOOH redox process of the nickel foam substrate.

The electrochemical response of F-doped KMn_4_(PO_4_)_3_ was evaluated using CV at different scan rates (2, 4, 6, 8 and 10 mV s^−1^) in 0.1 M KOH containing 5 mM ascorbic acid and 5 mM glucose individually, as shown in Fig. 5(e and f). The peak current increases as the scan rate rises. Additionally, the peak voltage shifts to higher values with increasing scan rates, indicating that the reaction is limited by how fast the analyte is consumed. This behavior is consistent with the irreversible nature of the oxidation processes and charge-transfer kinetics of the reactions. At higher scan rates, the oxidation reaction occurs over a shorter timescale and requires a higher overpotential, resulting in a positive shift of the anodic peak potential. Similar scan-rate-dependent positive shifts have been reported for the electrochemical oxidation of ascorbic acid and glucose and have been associated with irreversible oxidation and kinetic polarization.^61–64^ The insets of the graphs show a straight-line behavior between current density and the square root of the scan rate, which means that the reactions are mainly controlled by diffusion.^65,66^ In addition, the electrochemically active surface area (ECSA) was evaluated from the double-layer capacitance (*C*_dl_) using scan-rate-dependent CV measurements in the non-faradaic region (Fig. S2). The F-doped electrode exhibited a *C*_dl_ of 0.863 µF and an ECSA of 0.022 cm^2^, compared with 0.701 mF and 0.017 cm^2^, respectively, for the undoped electrode. This shows a 29% increase in the ECSA. The enhanced current response of the F-doped electrode is not only due to the increased electrochemically accessible surface area, but also associated with the improved intrinsic catalytic activity resulting from fluorine doping.^67^

Fig. 6(a and b) shows the CV graphs of the F-doped KMn_4_(PO_4_)_3_ electrode at a scan rate of 6 mV s^−1^ with two different concentration ranges of ascorbic acid in the millimolar range and micromolar range in 0.1 M KOH. As the concentration of ascorbic acid increases from 1 mM to 11 mM (Fig. 6(a)) and from 100 µM to 1700 µM (Fig. 6(b)), the oxidation peak current increases significantly. The anodic current response rises linearly with increasing ascorbic acid concentration, indicating the sensor's high sensitivity for its detection. Furthermore, the anodic potential shifts toward more positive values, which is attributed to diffusion-controlled mass transport.

**Fig. 6:**
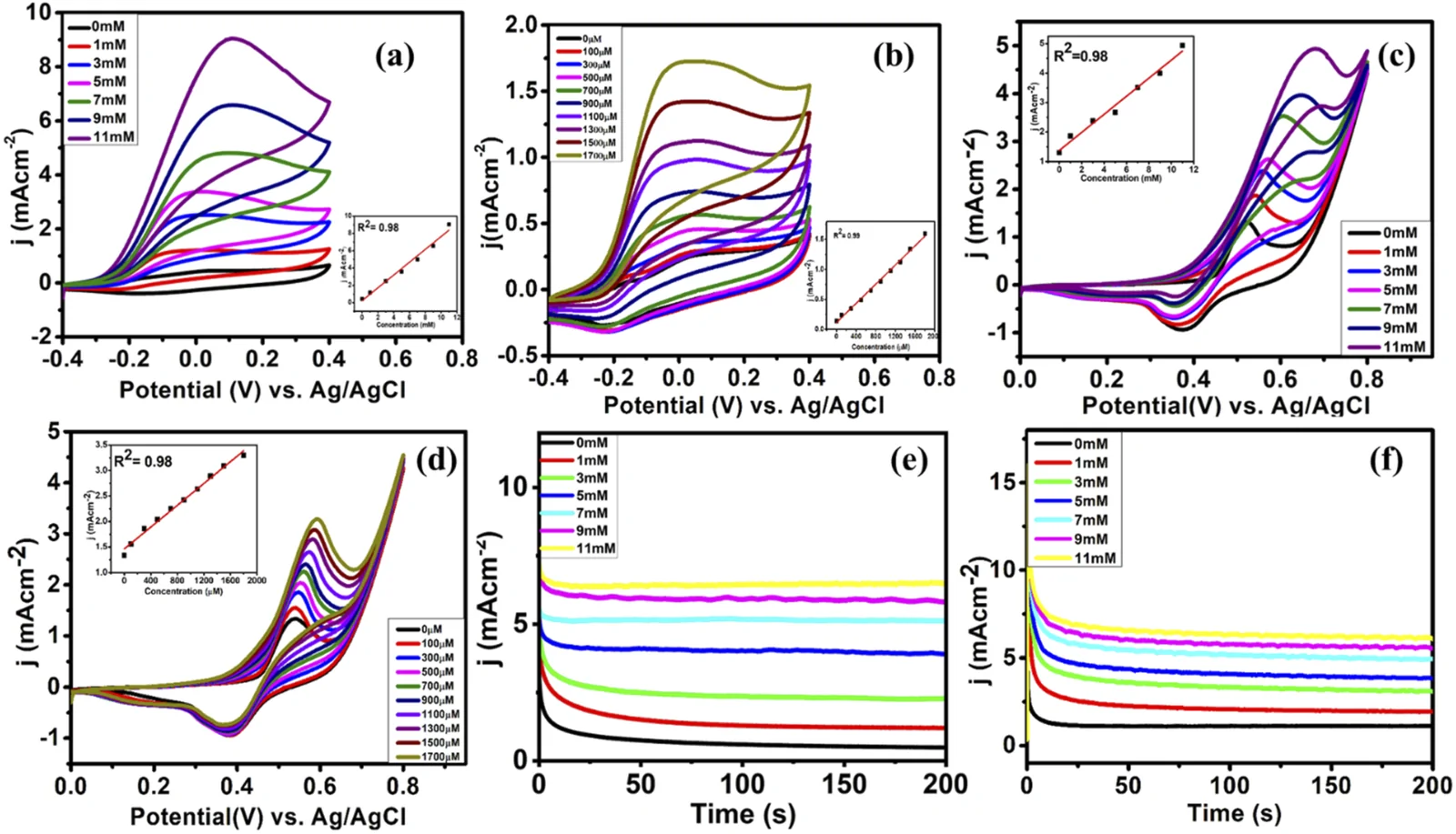
Effect of concentration. CV plots of (a) ascorbic acid and (c) glucose in the millimolar concentration range (insets: calibration plots). CV plots of (b) ascorbic acid and (d) glucose in the micromolar concentration range (insets: calibration plots). Chronoamperometric measurements at varying concentrations of (e) ascorbic acid and (f) glucose.

Similarly, Fig. 6(c and d) shows the sensor's linear response to various glucose concentrations at a scan rate of 6 mV s^−1^ in 0.1 M KOH, demonstrating the sensor's reliability and accuracy. The sensor response at millimolar concentrations is shown in Fig. 6(c), whereas the response at micromolar concentrations is presented in Fig. 6(d). The inset shows a straight-line relationship between the glucose concentration and the current density. The sensor's linear response towards different ascorbic acid and glucose concentrations was further verified by chronoamperometric measurements at their optimized potentials of 0.15 V for ascorbic acid and 0.60 V for glucose, as shown in Fig. 6(e and f).

During electrocatalytic oxidation of ascorbic acid and glucose at the sensor surface, F-doped KMn_4_(PO_4_)_3_ is expected to undergo initial oxidation in the presence of OH^−^ ions in the electrolyte, yielding F-doped K^+^Mn_4_^4+^(PO_4_)_3_(OH)_2_. This intermediate subsequently reverts to F-doped KMn_4_(PO_4_)_3_ while catalyzing the conversion of ascorbic acid (C_6_H_8_O_6_) and glucose (C_6_H_12_O_6_) into their oxidized forms, dehydroascorbic acid (C_6_H_6_O_6_) and gluconolactone (C_6_H_10_O_6_), respectively. The overall redox reaction is depicted in the following steps:F-doped KMn_4_^2+^(PO_4_)_3_ + 2OH^−^ → F-doped K^+^Mn_4_^4+^(PO_4_)_3_(OH)_2_ + 2e^−^.

F-doped KMn_4_(PO_4_)_3_ is oxidized by the OH^−^ ions in the electrolyte, as a result of which Mn changes to a higher oxidation state as follows:F-doped K^+^Mn_4_^4+^(PO_4_)_3_(OH)_2_ + C_6_H_8_O_6_ → C_6_H_6_O_6_ + F-doped KMn_4_^2+^(PO_4_)_3_ + 2H_2_O.

The OH^−^-formed intermediate converts ascorbic acid into its oxidized form, dehydroascorbic acid. Meanwhile, the intermediate is reduced back to the original form of F-doped KMn_4_(PO_4_)_3_.

Like ascorbic acid, a redox reaction of glucose occurs on the surface of the sensor material. The same steps are followed for glucose oxidation, where glucose is converted into gluconolactone. The overall proposed mechanism is summarized in the following steps:F-doped KMn_4_^2+^(PO_4_)_3_ + 2OH^−^ → F-doped K^+^Mn_4_^4+^(PO_4_)_3_(OH)_2_ + 2e^−^.

F-doped KMn_4_(PO_4_)_3_ is converted into an intermediate by reacting with OH^−^ ions from the electrolyte. During this step, Mn changes to a higher valent state as follows:F-doped K^+^Mn_4_^4+^(PO_4_)_3_(OH)_2_ + C_6_H_12_O_6_ → C_6_H_10_O_6_ + F-doped KMn_4_^2+^(PO_4_)_3_ + 2H_2_O.

The oxidized intermediate facilitates the oxidation of glucose to gluconolactone and is reduced back to F-doped KMn_4_(PO_4_)_3_. Such continuous electron transfer for both ascorbic acid and glucose between the sensor material and analytes enables electrochemical detection.

### 3.3. Detection and sensor performance metrics

Chronoamperometric measurements for the sensor material were conducted at three different potentials (0.10, 0.15 and 0.20 V) under continuous stirring, with incremental additions of 1 mM ascorbic acid after each 25 s interval in a 0.1 M KOH electrolyte solution, as shown in Fig. S3. The highest current density was observed at 0.15 V, indicating this as the optimal potential for ascorbic acid detection. Similarly, for glucose detection, chronoamperometric studies were performed at 0.55, 0.60, and 0.65 V, as shown in Fig. S4. The potential of 0.60 V was selected as optimal due to its superior current response.

Amperometric measurements were performed on the fabricated electrode at an optimized potential of 0.15 V with incremental additions of different molar concentrations of ascorbic acid in 0.1 M KOH, with continuous stirring, as shown in Fig. 7(a). Similarly, the staircase response was evaluated for glucose at 0.60 V, with incremental additions of varying molar concentrations of glucose under constant stirring in 0.1 M KOH, as depicted in Fig. 7(b). The electrode material exhibited a linear current response over concentration ranges of 10 µM to 14 mM for ascorbic acid and 50 µM to 14 mM for glucose. In the case of ascorbic acid, the sensor material showed a broader linear range than that for glucose. This may indicate fast kinetics for ascorbic acid. A zoomed-in view of the staircase response of F-doped KMn_4_(PO_4_)_3_ towards both ascorbic acid and glucose was examined. This view clarifies that the current response rises quickly after each ascorbic acid injection, reaching the steady-state value within 2 seconds, as shown in Fig. 7(c). In the case of glucose, the current response increases with each injection and approaches the steady-state value in 3 seconds, as shown in Fig. 7(d). This rapid current rise over a short period reflects the fast sensing capability and excellent sensitivity of the sensor material towards ascorbic acid and glucose detection. The F-doped KMn_4_(PO_4_)_3_ electrode material demonstrated high sensitivities of 800 µA mM^−1^ cm^−2^ for ascorbic acid and 1070 µA mM^−1^ cm^−2^ for glucose. It exhibited LOD and LOQ values of 0.07 µM and 0.20 µM for ascorbic acid and 0.10 µM and 0.30 µM for glucose, respectively. SI Tables S1 and S2 highlight different metal phosphate-based sensor materials and compare their performance with the synthesized F-doped KMn_4_(PO_4_)_3_ for the detection of ascorbic acid and glucose, respectively. This comparison indicates that F-doped KMn_4_(PO_4_)_3_ is a promising sensor material for both ascorbic acid and glucose detection (Table 1).

**Fig. 7:**
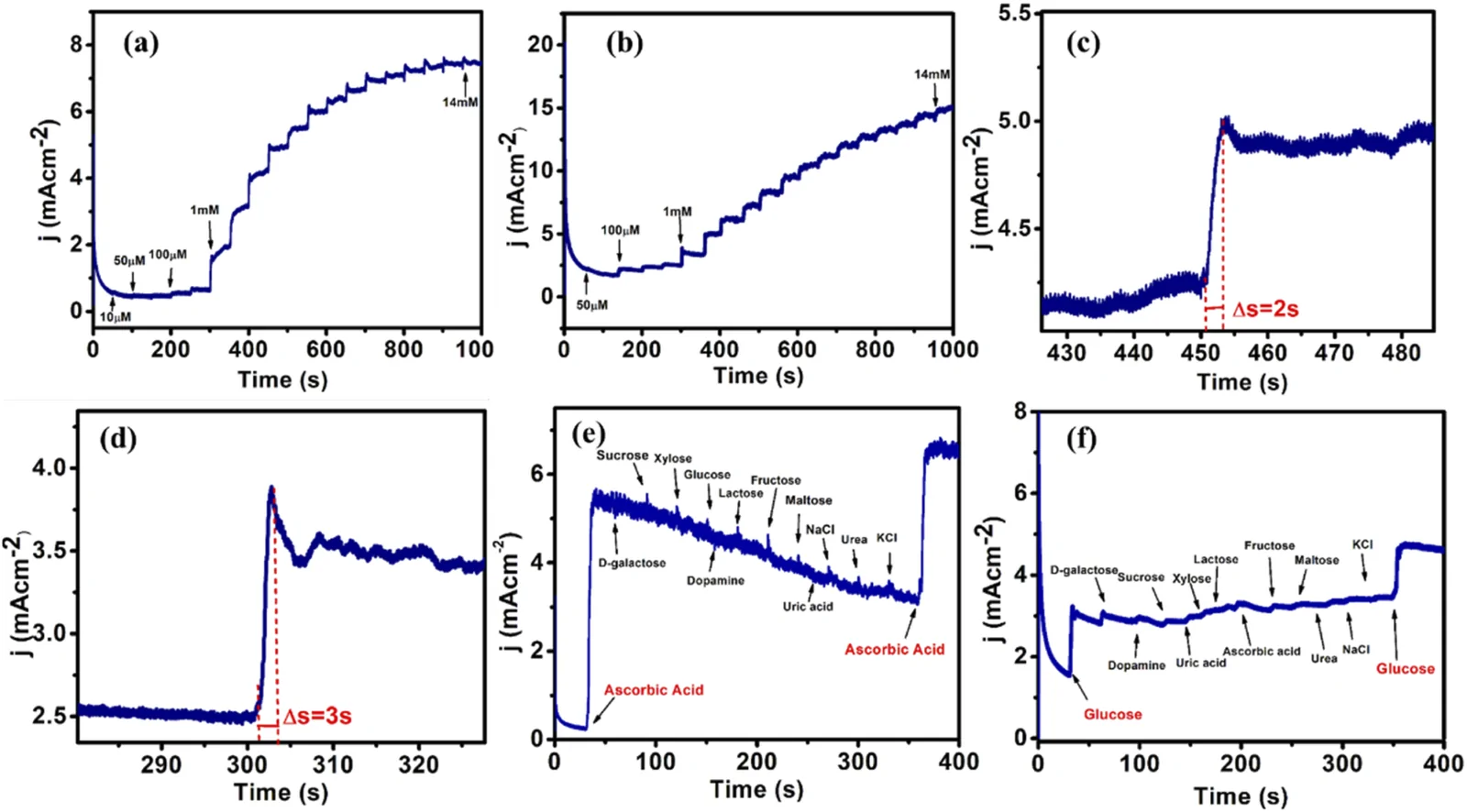
Chronoamperometric measurements. Staircase behavior of the F-doped KMn_4_(PO_4_)_3_ electrode for (a) ascorbic acid and (b) glucose. Magnified plots of the staircase response displaying the response time for (c) ascorbic acid and (d) glucose. Anti-interference study of (e) ascorbic acid and (f) glucose.

**Table 1:** Key analytical characteristics of ascorbic acid and glucose

Analyte	LOD (µM)	LOQ (µM)	Sensitivity (µA mM^−1^ cm^−2^)	Response time (s)	Linear range
Ascorbic acid	0.07	0.20	800	2	10 µM–14 mM
Glucose	0.10	0.30	1070	3	50 µM–14 mM

High selectivity is essential for non-enzymatic ascorbic acid and glucose sensors. This is because many easily oxidizable compounds present in the human body can interfere with detection at a fixed working potential due to similar electron-transfer behaviors. To test this, chronoamperometry was performed for ascorbic acid at 0.15 V with reference to Ag/AgCl in a 0.1 M KOH solution, with constant stirring. The effects of possible interfering compounds, such as d-galactose, sucrose, xylose, glucose, dopamine, lactose, fructose, maltose, uric acid, sodium chloride, urea, and potassium, were checked by adding 1 mM solutions of each interferent against a 100 µM solution of ascorbic acid. The obtained current responses of the interfering species were insignificant relative to the current response of ascorbic acid. This shows the high selectivity of the sensor material towards ascorbic acid at the specific potential of 0.15 V as shown in Fig. 7e. Furthermore, the anti-interference ability for glucose was tested at its optimized potential of 0.60 V in 0.1 M KOH under constant stirring by adding 1 mM solution of each interfering species, including d-galactose, dopamine, sucrose, uric acid, xylose, lactose, ascorbic acid, fructose, maltose, urea, sodium chloride, and potassium chloride, against a 100 µM glucose solution. The obtained current responses were either undetectable or negligible relative to the current response of glucose molecules. The F-doped KMn_4_(PO_4_)_3_ modified electrode showed excellent selectivity for detecting glucose, resisting interference from other interfering species at a potential of 0.60 V, as depicted in Fig. 7(f).

The stability of the modified electrode was examined by cyclic voltammetry and chronoamperometry. Fig. 8(a and b) presents the stability results obtained from CV measurements of 5 mM ascorbic acid (a) and glucose (b) at a scan rate of 6 mV s^−1^ in a 0.1 M KOH electrolyte using the F-doped KMn_4_(PO_4_)_3_ electrode over multiple cycles. Both graphs show a negligible decline in peak current and peak potential after 50 CV cycles. This slight decline is likely due to the continuous oxidation of ascorbic acid and glucose on the surface of the working electrode. This negligible change indicates good electrode stability and durability for long-term sensing applications. The stability of F-doped KMn_4_(PO_4_)_3_ for the detection of ascorbic acid and glucose was also evaluated by chronoamperometry in 0.1 M KOH containing 5 mM ascorbic acid and glucose at their optimized potentials of 0.15 and 0.60 V, respectively, *versus* Ag/AgCl. Fig. 8(c and e) represents the current responses for both ascorbic acid and glucose, respectively, while Fig. 8(d and f) shows the corresponding bar graphs. These findings indicate that the material maintains excellent electrochemical stability, retaining 92% and 93% of its initial current response for ascorbic acid and glucose, respectively, even after 50 days of testing. Fig. 8(g and h) shows the amperometric responses of five different electrodes of the sensor material (F-doped KMn_4_(PO_4_)_3_) prepared independently to 5 mM ascorbic acid and glucose in 0.1 M KOH at 0.15 and 0.60 V, respectively. The corresponding bar graphs show that the synthesized electrodes have high repeatability, with low relative standard deviation (RSD) values of 1.70% and 2.90% for ascorbic acid and glucose, respectively.

**Fig. 8:**
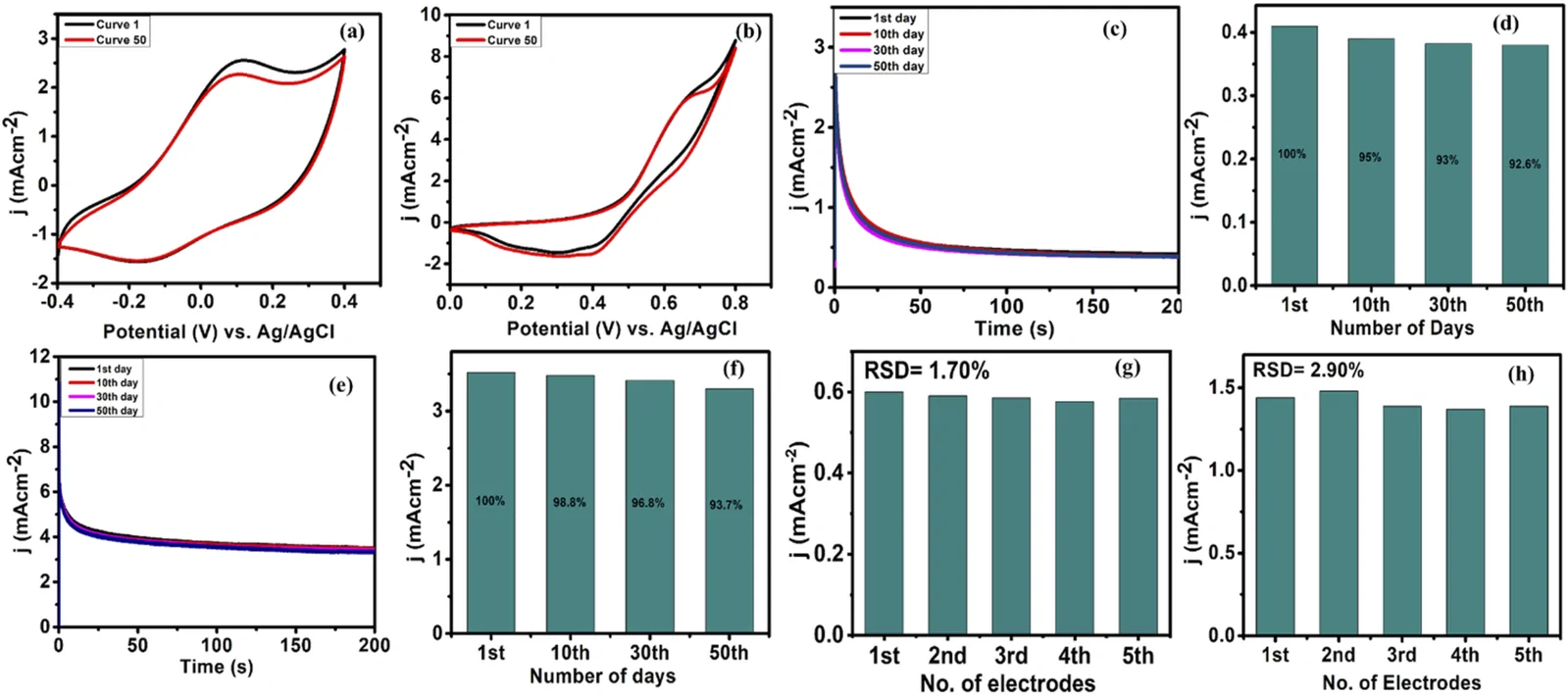
Stability test. 1st and 50th CV cycle stability comparison for (a) ascorbic acid and (b) glucose. Stability test of the prepared electrode over different time intervals for (c and d) ascorbic acid and (e and f) glucose. Reproducibility test of five different KMn_4_(PO_4_)_3_-based electrodes for (g) ascorbic acid and (h) glucose.

### 3.4. Real sample analysis

Amperometric measurements of the sensor towards real samples were performed in blood serum to assess its practical applicability. For this analysis, blood was first collected from a healthy person and then centrifuged to isolate blood serum. The serum was subsequently diluted 100-fold with 0.1 M KOH (1 : 100 v/v) to reduce matrix effects and bring the analyte concentrations within the linear ranges of the sensor material. Chronoamperometric readings were obtained at an optimized potential of 0.60 V for a blood serum sample spiked with a known amount of standard glucose in the KOH electrolyte. The normal range of glucose in blood serum is between 3.5 and 6 mM.^68^ The unknown amount in blood was determined using a linear calibration plot with a regression coefficient of 0.998. Percent recoveries were calculated, as mentioned in Table 2. These findings confirm that the F-doped KMn_4_(PO_4_)_3_-modified electrodes can monitor glucose levels in real-world samples.

**Table 2:** Results of the blood serum sample analysis of glucose and ascorbic acid

Blood serum sample	Content (mM)	Added (mM)	Determined (mM) (mean ± SD)	Recovery (%)	RSD (%)
Glucose	3.640	1	4.573 ± 0.021	93.33%	0.46
		3	6.513 ± 0.015	95.73%	0.23
		5	8.547 ± 0.078	98.13%	0.91
		7	10.457 ± 0.117	97.37%	1.12
Ascorbic acid	0.275	1	1.253 ± 0.015	97.77%	1.22
		3	3.160 ± 0.026	96.14%	0.84
		5	5.187 ± 0.015	98.22%	0.29
		7	7.223 ± 0.015	99.25%	0.21

The same analysis was also performed for ascorbic acid detection using the same serum sample at a potential of 0.15 V. In healthy humans, the normal range for ascorbic acid is 0.6–2 mg dL^−1^ (0.034–0.114 mM).^69^ Using the same standard addition method, different standards of ascorbic acid were spiked into blood serum samples for analysis. The calibration plot obtained was extrapolated to determine the unknown amount of ascorbic acid with a regression coefficient of 0.998. Percent recoveries were calculated, as shown in Table 2. These results show that the F-doped KMn_4_(PO_4_)_3_-modified electrode can effectively analyze ascorbic acid levels in actual physiological samples. For both analytes, the recovery experiments were performed as single measurements.

## 4. Conclusions

A co-precipitation method was used to synthesize F-doped KMn_4_(PO_4_)_3_, which serves as a high-performance, cost-effective, enzyme-free electrochemical sensing material. The F-doped KMn_4_(PO_4_)_3_ sensor exhibited enhanced electrocatalytic performance and excellent specificity for ascorbic acid at 0.15 V and for glucose at 0.60 V. The effectiveness of the sensor was confirmed through electrochemical impedance spectroscopy, cyclic voltammetry, and chronoamperometry. For ascorbic acid, F-doped KMn_4_(PO_4_)_3_ exhibited an LOD of 0.07 µM, an LOQ of 0.20 µM, and a sensitivity of 800 µA mM^−1^ cm^−2^. For glucose, the calculated LOD and LOQ were 0.10 µM and 0.30 µM, respectively, with a high sensitivity of 1070 µA mM^−1^ cm^−2^. Notably, the sensor achieved a response within just 2 s for ascorbic acid and 3 s for glucose. Moreover, the sensor material exhibited outstanding repeatability, with RSDs of 1.70% and 2.90% for ascorbic acid and glucose, respectively, and demonstrated sustained performance across prolonged operation. To examine its real-world applicability, the sensor was used to determine ascorbic acid and glucose levels in human blood serum samples, achieving maximum recoveries of up to 99% for both analytes.

## Conflicts of interest

There are no conflicts to declare.

## Supplementary Material

RA-OLF-D6RA06582A-s001

## Data Availability

The data that support the findings of this study are available from the corresponding author upon reasonable request. The data are not publicly available as they are part of ongoing research. Please address correspondence to me at ahaider@qau.edu.pk. Supplementary information (SI) is available. See DOI: https://doi.org/10.1039/d6ra06582a.
