# Supplementary material for: F-doped KMn4(PO4)3-based non-enzymatic electrochemical sensor for ascorbic acid and glucose: laboratory sensing and real-sample analysis
Source: RSC Adv. 2026 Sep 30;16(46):59415–27. doi: 10.1039/d6ra06582a (PMC13625059; doi:10.1039/d6ra06582a)
Supplement: Supplementary file 1 [file RA-016-D6RA06582A-s001.pdf]

Supplementary Information (SI) for RSC Advances.
This journal is © The Royal Society of Chemistry

## Supplementary Information

# F-doped $KMn_4(PO_4)_3$ Based Non-Enzymatic Electrochemical Sensor for Ascorbic Acid and Glucose: Laboratory Sensing and Real-Sample Analysis

**Hekmat Alham,[1] Sana Ahsen,[1] Taqdees Tariq,[1] Javeria Ahmed,[1] Maria Khalid,[2] Farhan Younas,[3] Talha Nisar,[4,5] Veit Wagner,[4] Aansa Khaliq,[6] Amin Badshah*,[1,7] Ali Haider*[1,7]**

[1] Department of Chemistry, Quaid-i-Azam University, Islamabad 45320, Pakistan

[2] H.E.J. Research Institute of Chemistry, International Center for Chemical and Biological Sciences, University of Karachi, Karachi, 75270, Pakistan

[3]Center for Interdisciplinary Research in Basic Sciences (CIRBS), International Islamic University Islamabad, Islamabad, Pakistan

[4]School of Science, Constructor University, 28759 Bremen, Germany

[5]Karlsruhe Institute of Technology (KIT), Institute for Applied Materials (IAM), Karlsruhe 76131, Germany

[6]Government Associate College For Women, Mandi Shah Jewana, Jhang.

[7]Pakistan Academy of Sciences, 3-Constitution Avenue, Sector G-5/2, Islamabad 44000, Pakistan

Corresponding Author: A.B. (aminbadshah@qau.edu.pk); A.H. (ahaider@qau.edu.pk)

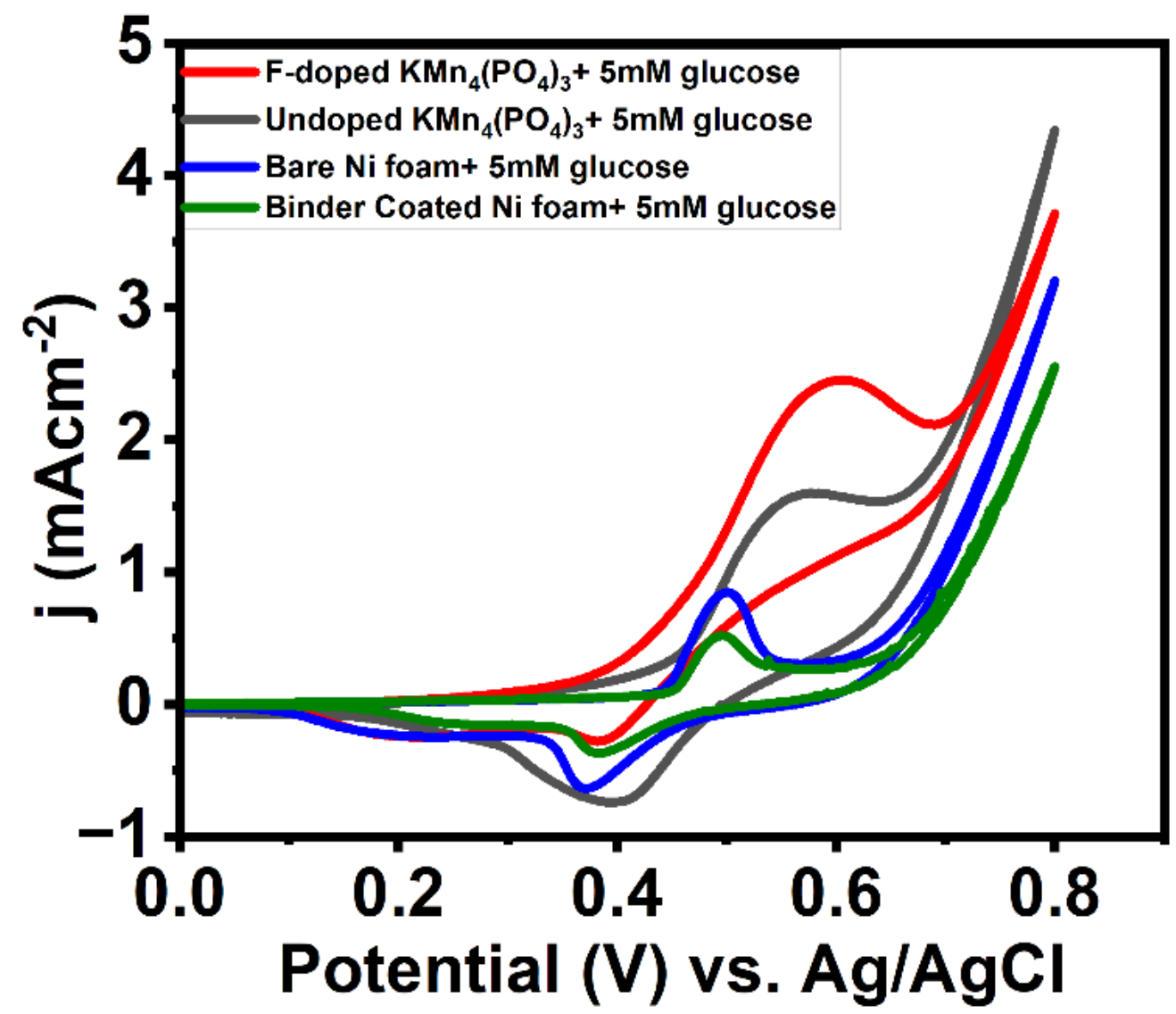


Figure S1. CV curves of bare nickel foam, binder-coated nickel foam, undoped $KMn_4(PO_4)_3$/Ni foam, and F-doped $KMn_4(PO_4)_3$/Ni foam towards glucose.

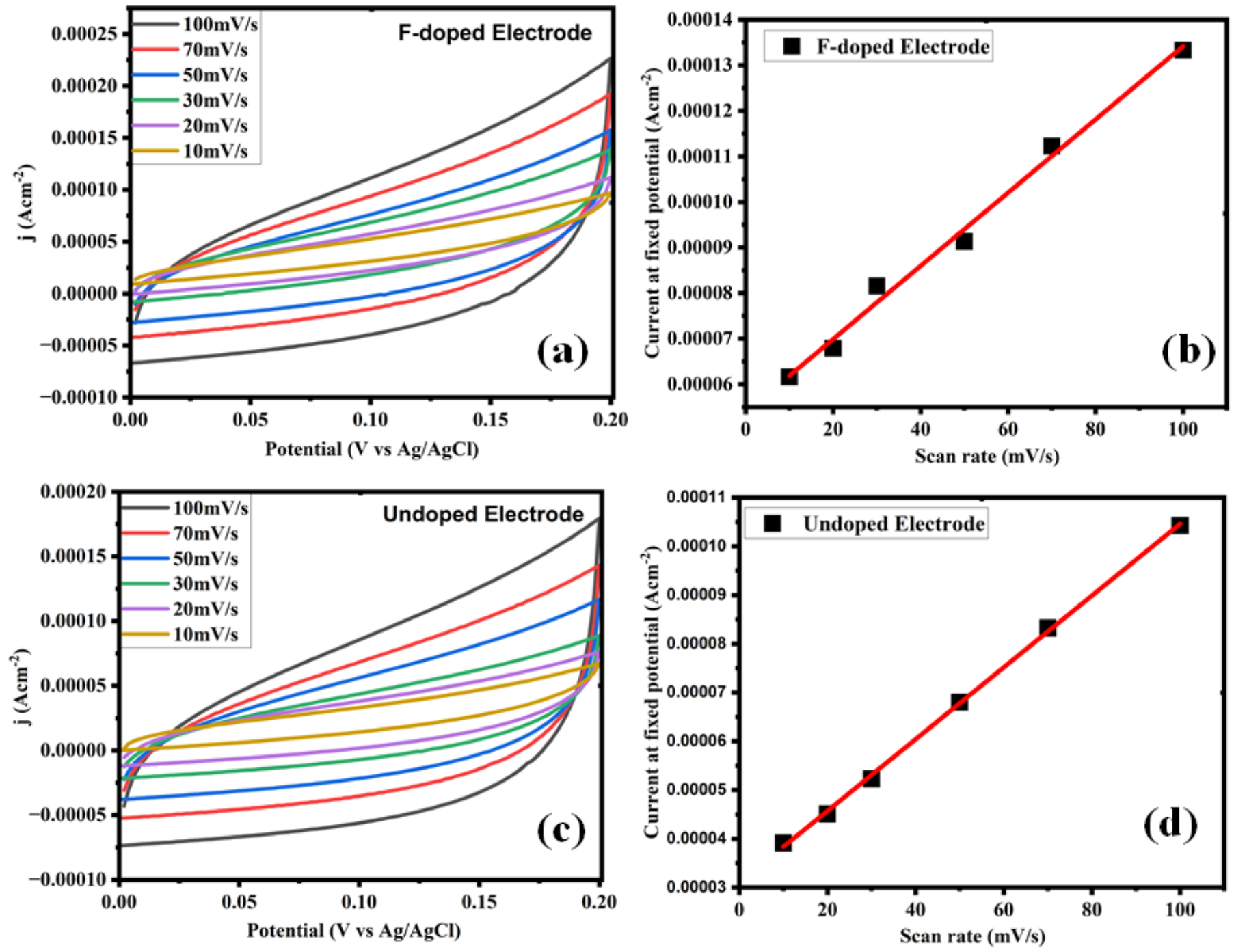


Figure S2. Cyclic voltammetry (CV) curves recorded at different scan rates in the non-faradaic region for (a) F-doped and (c) Undoped electrodes, and the corresponding linear plots of capacitive current versus scan rate for (b) F-doped and (d) Undoped electrodes used to determine the double-layer capacitance (Cdl) and electrochemically active surface area (ECSA).

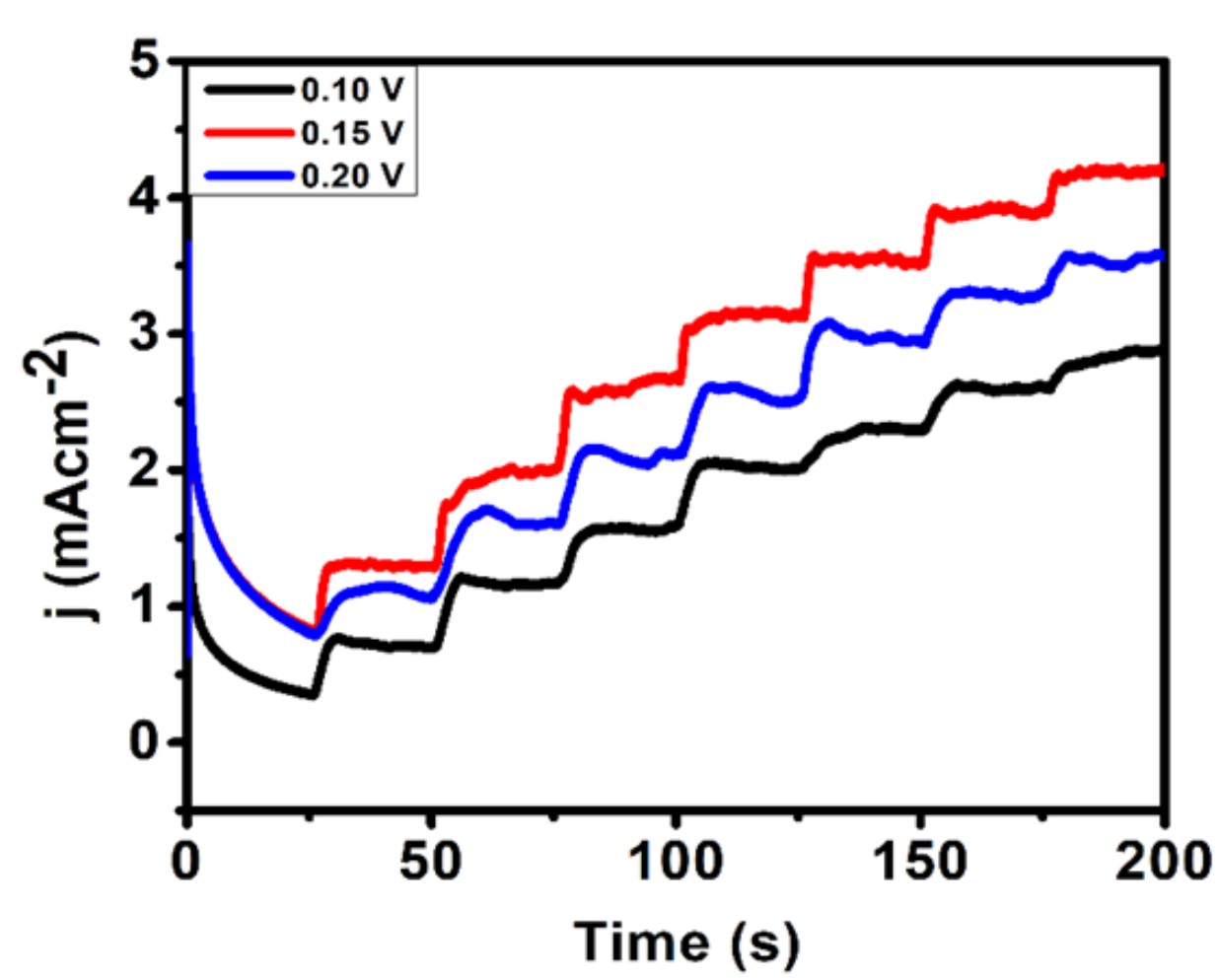

Figure S3. Voltage optimization for ascorbic acid detection

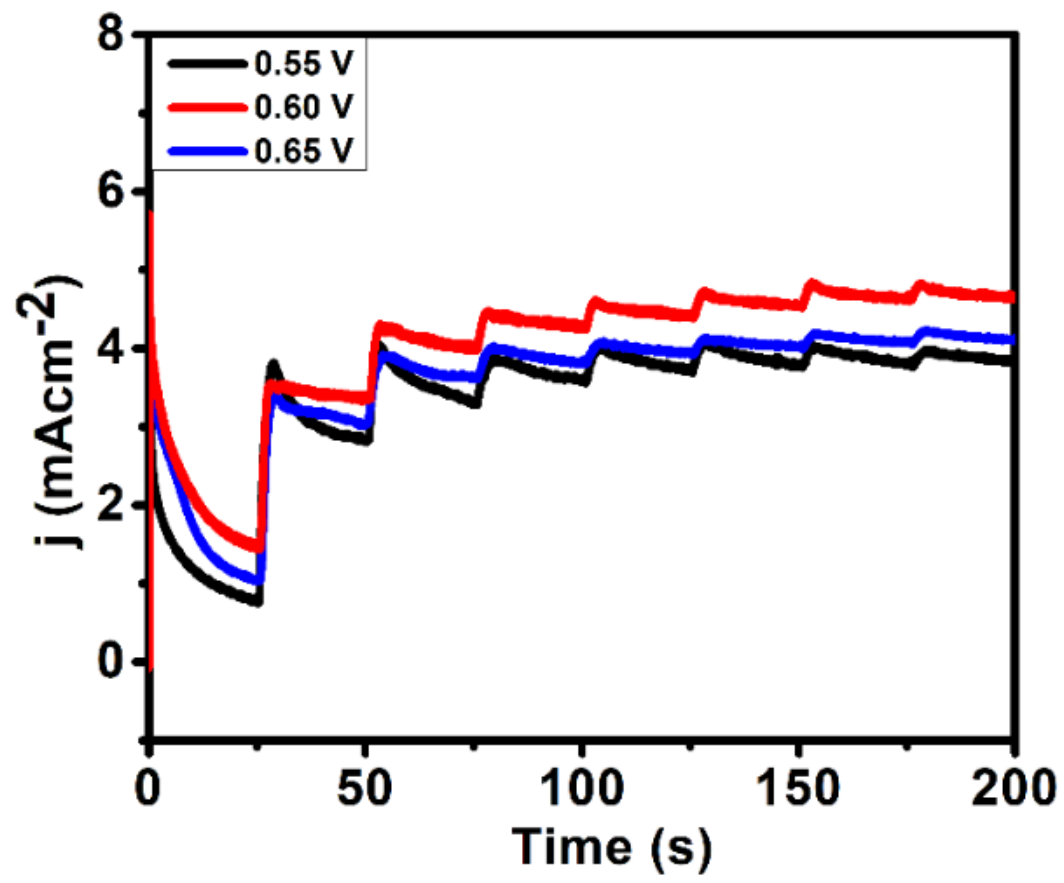


Figure S4. Voltage optimization for glucose detection

Table S1. Literature Comparison for Ascorbic Acid

| Materials | LOD | Linear Range | Sensitivity | References |
|---|---|---|---|---|
| Redox-active molybdophosphate film on a glassy carbon electrode (GCE) | 0.004μM | 1 μM to 1.5 mM | 219 μAmM$^{-1}$cm$^{-2}$ | [1] |
| Au-CEW-$Cu_3(PO_4)_2$-GO nanoflowers (HNFs) | 2.67 μM | 300 μM to 8 mM | 6.01 × 10$^{-6}$ μAmM$^{-1}$cm$^{-2}$ | [2] |
| Natural phosphate modified carbon paste electrode (CPE-NP) | 0.00144 $\mu$M | 1 μM to 1mM | - | [3] |
| Lithium cobalt phosphate (LCP) | 8.10 μM | 10 μM to 8mM | - | [4] |

| | | | | |
|---|---|---|---|---|
| Zirconium Phosphate (ZrP) | 24 μM | – | 11.7 μAmM$^{-1}$cm$^{-2}$ | [5] |
| GO/$TmPO_4$/GCE | 39 μM | 100 μM to 1 mM | – | [6] |
| Black phosphorus nanosheets (BPNS) | 0.0003 μM | 0.001 μM to $3.5\times10^{-5}$ mM | – | [7] |
| **F-doped $KMn_4(PO_4)_3$** | **0.07 μM** | **10 μM-14 mM** | **800 μAmM$^{-1}$cm$^{-2}$** | **This work** |

**Table S2. Literature Comparison for Glucose**

| **Materials** | **LOD** | **Linear Range** | **Sensitivity** | **References** |
|---|---|---|---|---|
| Cobalt Phosphate Nanostructures (CPNs) | 300 μM | 1000μM to 30 mM | 0.00790 $\mu$AmM$^{-1}$cm$^{-2}$ | [8] |
| $NH_4NiPO_4{\cdot}H_2O$ nanorods | 0.5 μM and 6 μM | 5 μM –2.1mM and 2400 μM-5.7mM | 459.4 μAmM$^{-1}$cm$^{-2}$ and 250.2 μAmM$^{-1}$cm$^{-2}$ | [9] |
| Ultrathin Nickel–Cobalt Phosphate 2D Nanosheets | 0.4μM | 2μM–4.47mM | 302.99μAmM$^{-1}$cm$^{-2}$ | [10] |
| $Ni_3(PO_4)_2$/CSs composite microspheres | 1.67 μM | 5 μM–2.5 mM and 2500 μM-7.5 mM | 480.153 μAmM$^{-1}$cm$^{-2}$ And 219.898 μAmM$^{-1}$cm$^{-2}$ | [11] |

| | | | | |
|---|---|---|---|---|
| $NH_4NiPO_4{\cdot}H_2O$ $Ni^{3+}$-rich surface | 0.009 $\mu$M | 1 $\mu$M – 1mM | 11,361 $\mu$AmM$^{-1}$cm$^{-2}$ | [12] |
| Flower-like cobalt phosphate micro sheet arrays supported on Ni foam | 1 μM | – | 3550 μA mM$^{-1}$ cm$^{-2}$ | [13] |
| Nickel phosphate electrocatalyst | 437 μM | 2.5 to 30 mM | 8312 μA mM−1 cm−2 | [14] |
| **F-doped $KMn_4(PO_4)_3$** | **0.10 μM** | **50 μM-14 mM** | **1070 μAmM$^{-1}$cm$^{-2}$** | **This work** |

## References


1. S. Liu, X. Jiang, and M. Yang, Electrochemical sensing of L-ascorbic acid by using a glassy carbon electrode modified with a molybdophosphate film, *Microchim. Acta,* 2019, **186**(7), 445.
2. J. Gao, H. Liu, K. Wu, J. Yan, and C. Tong, A novel nonenzymatic ascorbic acid electrochemical sensor based on gold nanoparticals-chicken egg white-copper phosphate-graphene oxide hybrid nanoflowers, *Nanotechnology,* 2021, **32**(32), 325504.
3. H. El Ouafy, T. El Ouafy, M. Oubenali, M. Mbarki, and M. Echajia, Electrochemical Evaluation of the Catalytic Effect of Natural Phosphate on the Oxidation of Aascorbic Acid: Analytical Application in Human Blood, Phys. *Chem. Res.,* 2021, **9**(1), 85-98.

4. Y. Xu, Z. Meng, Y. Meng, X. Li, and D. Xiao, Lithium cobalt phosphate electrode for the simultaneous determination of ascorbic acid, dopamine, and serum uric acid by differential pulse voltammetry, *Microchim. Acta,* 2021, **188**(6), 190.
5. D. R. do Carmo, and T. F. S. da Silveira, A new composite based on electroactive zirconium phosphate: morfology, structure and their behavior as a voltammetric sensor in the ascorbic acid detection, *J. Inorg. Organomet. Polym. Mater.,* 2019, **29**(4), 1205-1218.
6. H. Huang, Y. Yue, Z. Chen, Y. Chen, S. Wu, J. Liao, S. Liu, and H. r. Wen, Electrochemical sensor based on a nanocomposite prepared from $TmPO_4$ and graphene oxide for simultaneous voltammetric detection of ascorbic acid, dopamine and uric acid, *Microchim. Acta,* 2019, **186**(3), 189.
7. K. J. Tian, L. Hu, Y. P. Dong, and X. F. Chu, Application of black phosphorus nanosheets modified electrode for electrochemical determination of ascorbic acid, *Russ. J. Electrochem.,* 2019, **55**(12), 1221-1228.
8. P. P. Tomanin, P. V. Cherepanov, Q. A. Besford, A. J. Christofferson, A. Amodio, C. F. McConville, I. Yarovsky, F. Caruso, and F. Cavalieri, Cobalt phosphate nanostructures for non-enzymatic glucose sensing at physiological pH, *ACS Appl. Mater. Interfaces,* 2018, **10**(49), 42786-42795.
9. H. Yin, T. Zhan, J. Zhu, J. Chen, J. Gong, L. Wang, and Q. Nie, Urea assistant growth of ammonium nickel phosphate ($NH_4NiPO_4 \cdot H_2O$) nanorods for high-performance nonenzymatic glucose sensors, *J. Electroanal. Chem.,* 2019, **846**, 113150.
10. Y. Shu, B. Li, J. Chen, Q. Xu, H. Pang, and X. Hu, Facile synthesis of ultrathin nickel–cobalt phosphate 2D nanosheets with enhanced electrocatalytic activity for glucose oxidation, *ACS Appl. Mater. Interfaces,* 2018, **10**(3), 2360-2367.

11. T. Zhan, H. Yin, J. Zhu, J. Chen, J. Gong, L. Wang, and Q. Nie, $Ni_3(PO_4)_2$ nanoparticles decorated carbon sphere composites for enhanced non-enzymatic glucose sensing, *J. Alloys Compd.,* 2019, **786**, 18-26.

12. X. Wang, H. Jian, Q. Xiao, and S. Huang, Ammonium nickel phosphate on nickel foam with a $Ni^{3+}$-rich surface for ultrasensitive nonenzymatic glucose sensors, *Appl. Surf. Sci.,* 2018, **459**, 40-47.

13. X. Wang, M. Wang, S. Feng, D. He, and P. Jiang, Controlled synthesis of flower-like cobalt phosphate microsheet arrays supported on Ni foam as a highly efficient 3D integrated anode for non-enzymatic glucose sensing, *Inorg. Chem. Front.,* 2020, **7**(1), 108-116.

14. S. S. Pujari, S. A. Kadam, Y. R. Ma, S. A. Khalate, P. K. Katkar, S. J. Marje, and U. M. Patil, Highly sensitive hydrothermally prepared nickel phosphate electrocatalyst as non-enzymatic glucose sensing electrode, *J. Porous Mater.,* 2021, **28**(2), 369-381.
